# Assessment of Cardiovascular Parameters during Meditation with Mental Targeting in Varsity Swimmers

**DOI:** 10.1155/2016/7923234

**Published:** 2016-02-11

**Authors:** Tyvin A. Rich, Robert Pfister, John Alton, David Gerdt, Martin Baruch

**Affiliations:** ^1^Department of Radiation Oncology, University of Virginia School of Medicine, Charlottesville, VA, USA; ^2^Hampton University Proton Therapy Institute, 40 Enterprise Parkway, Hampton, VA 23666, USA; ^3^Department of Statistics, University of Virginia, Charlottesville, VA, USA; ^4^Center for the Study of Complementary and Alternative Medicine, University School of Nursing, Charlottesville, VA, USA; ^5^Empirical Technologies Corporation, P.O. Box 8175, Charlottesville, VA, USA

## Abstract

*Introduction*. Athletes who develop an immunosuppressed state because of intensive training get upper respiratory infections (URIs) and may respond to meditation. Reflective exercise (RE), a westernized form of Qigong, combines meditation, breathing, and targeted mental attention to an internal pulsatile sensation, previously shown to protect varsity swimmers from URIs during the height of training. We report here the evaluation of cardiovascular parameters measured during meditation combined with targeted imagery (interoception) in a cohort of varsity swimmers taught RE.* Methods*. Thirteen subjects were enrolled on a prospective protocol that used the CareTaker, a noninvasive cardiovascular monitor before, during, and after RE training. Questionnaires regarding targeted mental imagery focusing on a pulsatile sensation were collected. The cardiovascular parameters include heart rate, blood pressure, and heart rate variability (HRV).* Results*. Increased variance in the subjects' BP and HRV was observed over the training period of 8 weeks. In nine subjects there was an increased low frequency (LF) HRV that was significantly (*p* < 0.05) associated with the subject's awareness of the pulsatile sensation that makes up a basic part of the RE practice.* Summary*. These data support further evaluation of HRV measurements in subjects while meditating with mental imagery. This direction could contribute to better understanding of neurocardiac mechanisms that relate meditation to enhanced immunity.

## 1. Introduction

The stress of intense training of college swimmers can result in physiologic changes that predispose them to frequent upper respiratory infections (URIs). Studies have reported URI incidences of 42–45% during training periods ranging from 4 to 12 weeks [1–3] while other studies showed an 8-week incidence of respiratory illness of 84% [4]. These ill effects can be thwarted by the practice of reflective exercise (RE, a westernized form of Qigong) [5] shown by a reduction of URIs in varsity swimmers who maintained practice [6]. The enhanced athletic performance associated with combinations of breathing, relaxation, meditation, guided imagery, and slow movement exercises supports the notion that these practices can enhance immunity [7–10].

One mechanism that could be involved with the beneficial effects of RE is through modulation of the neuroimmune system reflected by activity of the vagus nerve [11]. In some studies vagal function has been assessed by measuring autonomic activity with heart rate variability (HRV) and this, in turn, has been found to be inversely related to levels of inflammatory markers [12]. Useful insight into this hypothesis is illustrated by the CARDIA study where over five thousand men and women of similar ages to the UVA swim team members were evaluated over 15 years with analog EKG signals in the resting position [13]. Digitized R-waves were used to calculate R-R intervals and heart rate for low and high frequency bands on 300-second epochs. These cardiac covariates and other measures of wellbeing (blood pressure, blood sugar, BMI, physical activity, and smoking history) were inversely related to levels of proinflammatory cytokines (C-reactive protein and IL-6) and possibly mediated by the vagal cholinergic anti-inflammatory pathway [14]. They support the practice of monitoring of HRV to look for a wellness marker especially with studying real time changes associated with exercises like RE.

We have previously evaluated thirty-two volunteers practicing meditation while heart rate and blood pressure were monitored with a noninvasive device (CareTaker can be viewed at http://www.empiricaltechnologies.com/). We found RE elevated cardiovascular variance of heart rate variability (HRV), blood pressure, and increased low frequency to high frequency ratios of HRV and blood pressure which we interpreted as autonomic modulation (unpublished observations). These findings indicated cardiovascular measurements of HRV and BP were feasible during meditation and guided us in the design of a subsequent trial. We report here a study with university varsity swimmers taught RE for the first time and monitored with the CT device before, during, and after training. We postulated that we would identify HRV correlates consistent with autonomic regulation and associated with inflammatory suppression that could account for the protection afforded swimmers taught this routine. We also assessed with questionnaires the individual's perception of a targeted internal pulse sensation (interoception) [15] that forms part of the RE method.

## 2. Methods

The study population consisted of thirteen varsity swimmers (5 males and 8 females) who were enrolled at the University of Virginia in their first (2), second (1), third (4), fourth (5), or graduate (1) year. One had taken a course in Qigong two years before, 2 did yoga but not regularly, one had taken a class in Buddhist meditation, and one had been previously treated with hypnosis for anxiety attacks and these were not permitted during RE training. One subject claimed to have frequent respiratory illnesses but the other 12 said they occurred rarely. The practice of RE was higher during the training period (4 to 5 times per week) and dropped to about once or twice per week at the last interview. Their initial systolic and diastolic blood pressure taken at rest just prior to meditation sessions ranged from 111 to 135/51 to 76 and varied a little over the course of training. Although anti-inflammatory, antiallergy, and decongestant medications were allowed to be taken freely by the subjects they were not used during RE training. All subjects signed a University of Virginia School of Medicine IRB approved consent form and the study was conducted according to IRB guidelines.

### 2.1. RE Training

RE combines three elements into a single 20- to 30-minute routine. The three elements are (1) reverse abdominal breathing technique, (2) a set of fluid slow movements coordinated with the breathing technique, and (3) sensory meditation in standing, seated, and supine positions. The swimmers learned the entire RE routine in 6 sessions, each lasting approximately 1 hour. Thereafter, the instructor met with the swimmers 6 additional half-hour practice sessions over a 2-week period.

Some forms of traditional Qigong use reverse abdominal breathing, but the type of breathing RE uses has 5 distinct, nuanced features: (1) breathing is “light,” exclusively through the nostrils; (2) the breathing cycle is approximately 3 seconds for inhalation and 3 seconds for exhalation; (3) lower abdominal muscles gently contract in sync with inhalation and relax in sync with exhalation; (4) teeth of the upper and lower jaw lightly touch together, which causes the mastoid muscles of the jaw to flex mildly; (5) the tongue is lifted up to fill the upper palate with the tip resting behind the front teeth.

The complete RE routine is a 3-phase process that takes approximately 30 minutes to complete. Phase 1 consists of doing the slow movement set, coordinated with the breathing technique, which takes approximately 4 minutes to complete. Phase 2 involves 5–8 minutes of sensory meditation in the standing position, with the hands placed alternately in front of the chest, the lower abdomen, or the head. During phase 3, the practitioner lies down in a supine position or else sits upright in a chair and then performs the breathing technique for approximately 20 minutes.

At some point during the 20-minute meditation, the practitioner may begin to feel an emergent vascular sensation (the targeted pulse) in the lower abdomen that ascends with the inhalation into the sinus cavity of the head. The practitioner may experience this sensation as a subtle pulsing in the sinus cavity or forehead region or as a general increase in cranial pressure. On the exhalation, the pulsing or pressure subsides, and the practitioner senses what appears to be the return of this decreased pulsing or pressure to the lower abdomen. The primary goal of RE practice is to acquire and sustain this emergent sensation throughout the full 20 minutes of phase 3. Once acquired, the sensation tends to become distinctly more vascular and less of a pressure phenomenon.

The estimates of the number of subjects needed to test our hypothesis were based on the experience about RE training in UVa swimmers [6] and a separate volunteer study conducted with the CT on volunteers previously taught RE (unpublished observations). From the first study the impression was that swimmers are well motivated to learn this meditation technique and that a majority of them continue to practice during the sports season. The CT data on volunteers that shows a robust shift in their cardiovascular dynamics during RE meditation have been used to estimate that a minimum of 11 subjects was recommended to reject the null hypothesis of no effect on the low frequency component of the R-R intervals (with probability of 0.95). This number was calculated under the assumption that current estimates of the mean differences between meditation and rest and their standard deviation were accurate.

### 2.2. CareTaker Measurements

Baseline CareTaker (CT) measurements were collected by the research nurse in a separate location from the RE training at specific appointment times during the day. The CT data were collected in the same time interval each day (e.g., 2 to 4 p.m.) to minimize circadian variation. The first data collection was prior to beginning RE, then at the end of the second week (after the first 8 RE sessions had taught the basics), and then at the end of the third and fourth weeks, and the last measurement was 4 weeks later.

The CT data were collected by a Velcro cuff placed on the base of the right thumb and after a brief pressure adjustment period, a baseline of 5 minutes was started. The subjects were asked to begin to meditate for 15 minutes. If the practice of meditation had not yet been taught, the subject was asked to sit quietly and to relax with eyes closed for 15 minutes.

## 3. Results

**Figure 1:**
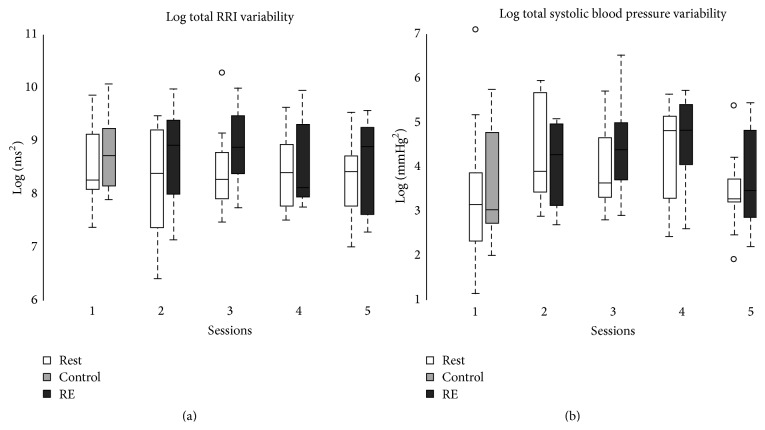
During session 1, subjects rested for 5 minutes followed by 15 minutes of uninstructed breathing. This 15-minute period is called the control period in the figures above. During sessions 2 through 5, subjects rested for 5 minutes, followed by 15 minutes of reflective exercises (RE).

The data summarizing the analysis of HRV and blood pressure are shown in Table 1 and show there is some increase in the RRI with meditation. There is less variation in the BP. The data in Figure 1 show that there is marked variation in RRI and blood pressure over the meditation sessions.

### 3.1. Signal Analysis

The CareTaker device records a series of blood pressure readings at 500 Hz. Heartbeats are detected by a process provided by ET, Inc. [16]. The CareTaker unit has been directly compared to intra-arterial catheter measurements and to EKGs in hospitalized patients and shows a high one to one correlation with the heart beat measured by those conventional methods (EmpiricalTechnologies, unpublished data, personal communication). In the analysis used here, the process applies a smoothing algorithm to the blood pressure series, indicating peaks above a moving threshold. For each of the heartbeats, the systolic blood pressure is calculated. The interbeat intervals are calculated as the differences between the peak times. Additionally, outliers in the interbeat interval series were deleted on a case-by-case basis to account for potential missed heartbeats or other aberrations. Outliers in the systolic blood pressure series were deleted if they exceeded 3 times a median absolute deviation from the median within a minute-long window.

Fourier spectral power was calculated by applying the Lomb periodogram method for unevenly sampled data. R Code for these calculations was provided by The Stowers Institute for Medical Research [17, 18]. Low frequency power (0.04–0.15 Hz) and high frequency power (0.15–0.4 Hz) were calculated as the area under the Lomb spectral density over the respective frequency bands, multiplied by total sample variance. For presentation purposes, these values are presented in log-scale (R development core team, 2008) [18].

As with many longitudinal studies, the data contains missing values due to missed appointments and in 4 cases dropping out of the study before the 3rd measurement. This is a potential source of bias, but it is assumed that the missing values are missing completely at random.

**Figure 2:**
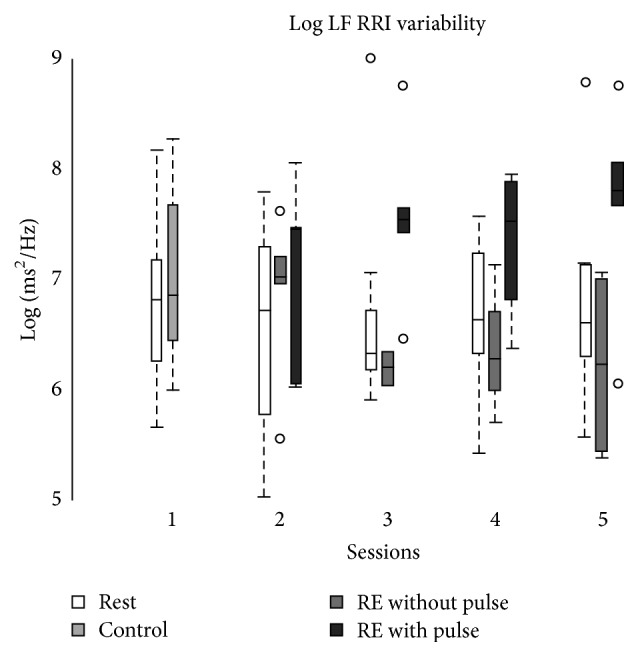
During session 1, subjects rested for 5 minutes, followed by 15 minutes of uninstructed breathing. This 15-minute period is called the control period in the figures above. During sessions 2 through 5, subjects rested for 5 minutes, followed by 15 minutes of reflective exercises (RE).

Kendall's Tau-b correlation test is performed to test for association between a subjective “pulse” sensation and various statistics including the LF HRV component. The relationship between these variables is shown as a boxplot in Figure 2 and Table 2. The data indicate that there is a statistically significant correlation with the sensation of the pulse and LF HRV.

## 4. Discussion

RE meditation has previously been shown to protect varsity swimmers from URIs at the height of seasonal incidence where aggregated cold/flu symptoms were significantly reduced by RE when practiced at least once per week [6]. The RE practitioners did not differ from nonpractitioners in demographic or lifestyle characteristics, medical history, supplement or medication use, or belief in RE. Also, like our data presented here, not all swimmers acknowledged feeling of the pulse sensation. In the present study we observed increased variance of blood pressure and HRV during an eight-week training period which covered approximately the same time period of intense training and high seasonal incidence of URI as the previous RE swim study. High variability of HRV cardiovascular parameters is associated with healthy conditions when compared to the blunted values that are seen with chronic disease states like heart failure and a variety of pathologic conditions [19]. The HRV changes found in our subjects in a period as short as 6 to 8 weeks of RE practice resemble observations of beneficial effects that can accrue from a meditation technique like the relaxation response [20].

The spectral analysis of the HRV in our swimmers shows that the LF component increased with training and is consistent with previous observations made in volunteers who practiced RE (unpublished observations) and in seasoned meditators [21]. The LF HRV parameter detected in both newly taught and seasoned RE practitioners suggests that there is a high level of autonomic balance and that modulation by the cardioneuroimmune network may be related to the protection from URIs in practicing swimmers. Although some have suggested that neuroimmunity is mainly bolstered by the HF component (the parasympathetic network) of the HRV, a recent review of the correlations of HRV and inflammation suggests the opposite [12]. HRV time domain indices like SDNN, SDNN index, and SD ANN and LF frequency-domain measures have significant associations with lower levels of inflammatory markers. Traditional “vagal measure” like LF HRV, a complex measure reflecting both parasympathetic and sympathetic activity, is the more commonly associated measure linked to low inflammatory markers [12]. This observation coupled with a newer understanding of the mechanisms of efferent vagal signaling with a lung inflammation model shed new light on the anti-inflammatory role of the vagus at the biomolecular level [22]. More germane to our clinical picture, the use of vagal stimulation (afferent pathways) has been shown to suppress inflammatory responses [23, 24]. The importance of vagal signaling in the inflammatory pathway is not disputed, as these accumulating data raise questions about the contribution of voluntary vagal stimulation through meditation and mental targeting in regulating immunity by a cardioneuroimmune mechanism.

## Figures and Tables

**Table 1:** 

Session	Task	RRI (ms)	RRI total variance (ms^2^)	Systolic BP (mmHg)
1	Rest	1004	5972	120.46
	Control	1013	7679	119.09

2	Rest	911	5669	120.00
	Meditation	920	8313	118.44

3	Rest	923	7934	134.81
	Meditation	928	9959	133.24

4	Rest	911	6001	129.77
	Meditation	926	7903	125.13

5	Rest	925	5596	122.91
	Meditation	941	7069	121.44

RRI = R-R interval; LF = normalized low frequency; HF = normalized high frequency; BP = blood pressure.

**Table 2:** 

Session	Subjects	Pulse	*p* value
2	10	5	0.301
3	9	6	0.026
4	7	4	0.039
5	9	5	0.025

Subjects refers to the number of subjects available to record data during each session. Pulse refers to the number of subjects who recorded feeling a pulse while performing the RE exercises. The *p* values are for Kendall Tau-b correlation tests of the subjective pulse response and the increase in LF power.
